# Genome-Wide Analysis of the Cyclin Gene Family and Their Expression Profile in *Medicago truncatula*

**DOI:** 10.3390/ijms21249430

**Published:** 2020-12-11

**Authors:** Juan Meng, Mengdi Peng, Jie Yang, Yiran Zhao, Junshu Hu, Yuntao Zhu, Hengbin He

**Affiliations:** Beijing Key Laboratory of Ornamental Plants Germplasm Innovation and Molecular Breeding, National Engineering Research Center for Floriculture, Beijing Laboratory of Urban and Rural Ecological Environment, School of Landscape Architecture, Beijing Forestry University, Beijing 100083, China; juanmeng@bjfu.edu.cn (J.M.); mengdi2020@bjfu.edu.cn (M.P.); jieyang@bjfu.edu.cn (J.Y.); yiranzhao@bjfu.edu.cn (Y.Z.); junshuHu@bjfu.edu.cn (J.H.); yuntao_zhu@bjfu.edu.cn (Y.Z.)

**Keywords:** cell cycle, cyclins, evolutionary analysis, expression profiling, legume, nodules, phylogenetic analysis

## Abstract

Cyclins, together with highly conserved cyclin-dependent kinases (CDKs), play an important role in the process of cell cycle in plants, but less is known about the functions of cyclins in legume plants, especially *Medicago truncatula*. Our genome-wide analysis identified 58, 103, and 51 cyclin members in the *M. truncatula*, *Glycine max*, and *Phaseolus vulgaris* genomes. Phylogenetic analysis suggested that these cyclins could be classified into 10 types, and the CycB-like types (CycBL1-BL8) were the specific subgroups in *M. truncatula*, which was one reason for the expansion of the B-type in *M. truncatula*. All putative cyclin genes were mapped onto their own chromosomes of each genome, and 9 segmental duplication gene pairs involving 20 genes were identified in *M. truncatula* cyclins. Determined by quantitative real-time PCR, the expression profiling suggested that 57 cyclins in *M. truncatula* were differentially expressed in 9 different tissues, while a few genes were expressed in some specific tissues. Using the publicly available RNAseq data, the expression of Mtcyclins in the wild-type strain A17 and three nodule mutants during rhizobial infection showed that 23 cyclins were highly upregulated in the nodulation (Nod) factor-hypersensitive mutant *sickle* (*skl*) mutant after 12 h of rhizobium inoculation. Among these cyclins, six cyclin genes were also specifically expressed in roots and nodules, which might play specific roles in the various phases of Nod factor-mediated cell cycle activation and nodule development. Our results provide information about the *cyclin* gene family in legume plants, serving as a guide for further functional research on plant cyclins.

## 1. Introduction

Cell division is the most basal process in biological growth and development. Any discussion of the role of cell division in plant development and growth requires a thorough understanding of the basic machinery that controls the cell cycle [1]. Progression of the eukaryotic cell cycle is primarily controlled by a kinase protein family known as the cyclin-dependent kinases (CDKs). Catalytic activities of CDKs were regulated in a complex manner, including cyclin binding and activation; CDK phosphorylation/dephosphorylation; direct binding of the CDK inhibitor protein (CKI) and CDK subunit (CKS); proteolysis; intracellular trafficking; and homologs of retinoblastoma protein (Rb), E2F transcription factors (E2F), and the dimerization partner (DP) pathway [1,2,3,4,5,6,7,8]. Among these protein factors, CDKs and cyclins are the most important cell cycle regulation proteins [9]. The first cyclin protein was discovered from sea urchin oocytes [10], after which more cyclins, CDKs, CKIs, and E2F transcription factors were identified in both animals and plants [7,11,12]. Cyclins complex with CDKs to control the activity, substrates, and subcellular localization of CDKs [7]. Different CDK–cyclin complexes phosphorylate a plethora of substrates at the key G1-to-S and G2-to-M transition points, triggering the onset of DNA replication and mitosis, respectively.

In animals, at least 13 classes of cyclins have been described (A to L, T, and UNG2-type) [13,14,15]. Since the first plant cyclin gene was cloned in soybean [16], more cyclins have been found in various plants [11,12]. A previous study indicates that plant cyclins can be classified into 10 groups, but there are only eight ancestral genes in the most recent common ancestor (MRCA) of extant green plants [17]. Forty-nine cyclins have been identified in the *Arabidopsis thaliana* genome, which were assigned to A-, B-, C-, D-, H-, L-, T-, U-, SDS-, and J18-type. The research suggests that D-, T-, U-, SDS-, and J18-type are the new subgroups in *Arabidopsis*, which are the plant-specific types [15]. Forty-nine cyclins forming nine families have been detected in the rice (*Oryza sativa)* genome, and F-type cyclins are specific to monocots [18]. Previous studies have identified 59 cyclins in the maize (*Zea mays*) genome and 52 in tomato, and have predicted 45 in poplar (*Populus trichocarpa*) [19,20]. Distinguished by the classes of organisms, cyclins have also been classified into M-cyclins and G1-cyclins according to the phase in which they function in the cell cycle. G1 cyclins include the C-, D-, E-, and G-type to regulate the G1-to-S transition. M cyclins, including the A- and B-type, function in S-to-M phase control, G2-to-M transition and intra-M-phase control [1,21]. According to the systematic evolutionary, cyclins can be divided into three major groups: group I, II and III, with different functions [22].

All cyclins possess a highly conserved N domain, or “cyclin box”, and a less conserved C domain that may not be necessary for cyclin functions [15,23,24]. In both animals and plants, most cyclins are expressed and function in the phase of the cell cycle. C13-1 and S13-6, the A-type and B-type cyclins cloned earliest in soybean, are expressed in the somatic embryo and leaves and roots of soybean seedlings, respectively [16]. Different A-type cyclins accumulate from early G1 to S phase and until the middle M phase of the cell cycle in plants [25]. For instance, in *Catharanthus roseus*, CYS mRNA, an A-type-like cyclin, accumulates at the onset of S phase and disappears early in G2 phase [26]. The overexpressed tobacco CYCA3;2 cyclin shows ectopic cell division and delayed differentiation, correlating with an increase in expression of S phase-specific genes and CYCA3;2-associated CDK activity [27]. These data suggest that A-type cyclins are expressed throughout the entire cell cycle and may have different functions in plants. B-type cyclins are expressed within a narrow time window in both G2-to-M transition, intra-G2-phase and intra-M-phase control [1,26]. In addition, ectopic expression of both Arath;CYCB1;1 and Oryza;CYCB2;2 accelerates root growth [28,29].

Besides the A- and B-type cyclins, D-type cyclins are one of the largest and plant-specific groups in plants. In *Arabidopsis*, 10 CYCDs have been identified, which fall into seven sub-groups (CYCD1 to CYCD7) [15]. In *Arabidopsis*, the D-types are prominently expressed and interact with CDKs to participate in G1-to-S phase. For example, the D-type cyclin CYCD3;1 is limiting for G1-to-S phase transition [30]. CYCD4;1 is expressed during lateral root primordium formation and interacts with CDC2aAt in starved suspension cultures upon mitogenic stimulation, indicating that the formation of a complex between these two partners is important for the resumption of cell division activity [31]. In addition, CYCD4 controls cell division in the stomatal lineage of the hypocotyl epidermis [32]. In tobacco, CYCD2 and CYCD3 show distinct effects on the structural organization of the shoot apical meristem. Ectopic expression of Arath-CYCD2 and Arath-CYCD3 affect the number, size and position of cells in the L1, L2, and L3 layers [33,34]. Genome-wide mume (*Prunus mume*) CYCDs include *PmCYCD1;2*, which is dependent on the presence of sucrose and induced by hormones. Stimulated by naphthyl acetic acid (NAA), *PmCYCD3;1*’s induction is increased when sugar is together with hormones [35]. Similarly, Some D-type cyclins probably act as key switches in triggering hormonal effects, such as CYCD3;1 and CYCD2;1 [30,36]. Additionally, excluding these CYCA, CYCB and CYCD-type cyclins, other cyclins in plants have not been systematically studied with respect to their function.

In *Medicago*, the two cyclins *cycMs1* and *cycMs2* were first cloned in *Medicago sariva*, showing that *cycMs2* can be classified as a type-B cyclin [37]. Then, more cyclins were found in *Medicago*. Another *cycMs3* gene, identified in A-type cyclins, is induced in the Go-to-G1 transition [38]. The D-type cyclin *cycMs4,* expressed predominantly in roots, plays a role in the G1-to-S transition, providing a model to investigate the plant cell cycle at the molecular level [39]. Additionally, A2-type cyclin is upregulated by auxin and involved in meristem formation [40,41]. Apart from its function in the cell cycle, some cyclin members respond to the requirement for auxin signaling in rhizobial infection and regulate the course of nodule organogenesis in *Medicago* [42,43]. Several cyclin genes are expressed in the nodulation (Nod) factor-susceptible root zone and at different stages of nodule development, such as A2-type cyclin *CycA2;1*(*cycMs3*)*,* B-type *cycB2* and D-type *CycD3;1(cycMs4).* These cyclins might be involved in the various phases of Nod factor-mediated cell cycle activation [42]. However, most cyclins isolated in alfalfa belong to *M. sariva*, and less systematic research has been conducted to examine the expression patterns of the majority of other legume species such as *M. truncatula* cyclin genes. Detailed phylogenetic analysis and classification of legume cyclins are still lacking. In this paper, we describe an extensive search for the legume cyclin gene family and phylogenetic analyses of these proteins. A total of 58, 103, and 51 members were identified in the *M. truncatula,* soybean (*G. max*) and common bean (*P. vulgaris*) genome, respectively. Furthermore, we report results from phylogenetic studies, classifications and names of subfamilies, gene structures and protein conserved motifs, identification of chromosomal locations, duplication events, cis-acting element prediction and expression analysis of cyclin genes. The expression profile indicated that some Mtcyclins have potential roles in Nod factor-mediated cell cycle activation and nodule development, which should provide genome-level insights into cyclin genes.

## 2. Results

### 2.1. Identification of the Cyclin Gene Family in Legume

To identify cyclin genes in three legume species, namely, *M. truncatula*, soybean, common bean genomes, hidden Markov models (HMMs) were used to query the whole genome by using the cyclin_N and cyclin_C domains in the Pfam database, respectively. We initially searched a total of 145 members in *M. truncatula,* 293 in soybean and 89 in common bean. Then, after removing the repeat transcriptions, there were 64 members in *M. truncatula,* 112 in soybean and 54 in common bean (Table S2). Previous studies have indicated that cyclins contain a conserved 250-amino-acid region called the cyclin core, which contains two domains: cyclin_N and cyclin_C [24]. The cyclin _N domain is more conversed than the cyclin _C, so we decided that candidates containing at least one cyclin_N domain were considered “true” cyclins [15]. Six members that did not contain the cyclin_N domain were discarded, and 58 members were obtained in *M. truncatula* (Table S2). By constructing a phylogenetic tree of these 58 members with *Arabidopsis* cyclins, a member with a sequence length that was too short compared with the other cyclins was found and removed. By adding one member we found from the previous study, a total of 58 putative cyclin genes were finally obtained. Similarly, 9 and 3 non-conforming members were deleted from soybean and common bean, and 103 and 51 cyclin genes were obtained, respectively (Table S2). In *M. truncatula*, all 58 cyclin proteins contain the cyclin_N domain, and 38 of them also have the cyclin_C domain, whereas the remaining 20 only have the cyclin_N domain. Detailed information on the specific identification process and the number of cyclins, subfamilies, conserved domains and amino acids is provided in Table S2.

### 2.2. Phylogenetic Tree Analysis and Classification of Cyclin Genes

**Figure 1:**
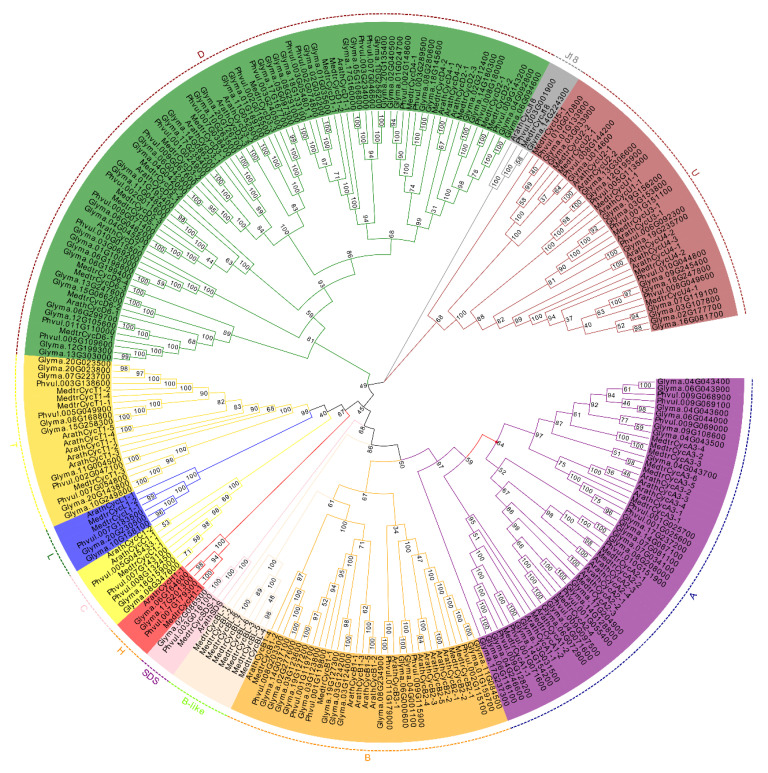
Phylogenetic tree analysis of cyclins retrieved in *Medicago truncatula*, soybean, common bean and *Arabidopsis*. The complete protein sequences for 261 cyclins were aligned by ClustalW, and the phylogenetic tree was constructed using MEGA 7.0 by the neighbor-joining method with 1000 bootstrap replicates. All cyclins were classified into 10 distinct groups based on the nomenclature of Arabidopsis cyclins (from A to D, H, L, T, U, SDS and J18) and were distinguished by different colors.

It is known that the *Arabidopsis* genome encodes at least 49 cyclins, which have been divided into 10 types based on sequence and function analysis [15]. To determine the evolutionary relationship and classification of these legume cyclins, phylogenetic analysis was performed for cyclins from three legume species and *Arabidopsis* genomes by using the neighbor-joining method and maximum likelihood method (Figure 1 and Figure S1). The putative 58 *M. truncatula* cyclin genes, 103 soybean cyclins and 51 common bean cyclins were classified into 10 types according to the phylogenetic tree with *Arabidopsis* cyclins (Figure 1). Phylogenetically, A- and B-type cyclins are more closely related to each other than to other types. Additionally, the SDS-type was grouped closer to the A- and B-type. The C-, L- and H-type formed a small independent clade, which was closer to the T-type clade, consistent with rice cyclins [18]. J18-type cyclins formed a separate clade and were not closely related to other types of cyclins. There were 73 members in D-type cyclins, forming the largest cluster in the four-plant cyclin family (Figure 1). Moreover, we found that *M*. *truncatula* cyclins had a specific clade in CycB, which lacked close homologs in *Arabidopsis*, soybean and common bean cyclins. Therefore, we designated these as members of the MedtrCycB-like type, which contained eight members. The results indicated that these cyclin genes might play an important and specific role in developmental and physiological processes in *M*. *truncatula*.

To identify the members of each cyclin type, the number of each group cyclins from *Arabidopsis*, *M. truncatula*, soybean and common bean was quantified; the cyclins’ content is listed in Table 1. The results suggest that CycA and CycB, both including 11 gene members, represented the largest groups in *Arabidopsis*, but it was CycD that contained the most gene members in legume plants. This result indicated that CycD was one of the largest subfamilies in legume plants. In contrast, CycJ18, as the smallest group, contained only one member in each species. Except for the CycB and CycJ18, there were approximately twice as many members in most subgroups of soybean compared with *M*. *truncatula*. Similarly, there were approximately twice as many members in common bean compared with *M. truncatula,* apart from CycC, CycJ18 and CycSDS. In *M. truncatula* and common bean, except for the B-type, the distribution trend of the number of cyclins in other groups was similar.

To further study the classification of the *M. truncatula* cyclin subgroups and names, phylogenetic analysis was performed for cyclins from the *M. truncatula*, soybean, common bean and *Arabidopsis* genomes (Figure 1). The *M. truncatula* cyclins were named based on the phylogenetic relationships determined by the common branches with Arabidopsis cyclins. The MedtrCycD-type cyclin genes can be divided into six subgroups corresponding to CycD1, CycD2, CycD3, CycD5, and CycD6. MedtrCycA-type consists of CycA1, CycA2, and CycA3 cyclins. MedtrCycU-type contain CycU1 to CycU4 cyclins, and C-, H-, L- and T-type have one subgroup of CycC1 and CycT1, respectively. On the contrary, in *M. truncatula,* eight cyclins formed a separate clade closely related to A-, B- and SDS-type. Phylogenetic analysis with *Arabidopsis* and human cyclins showed that they still clustered with B-type cyclins (data not shown). Thus, we named the cyclins in this clade as CycB-like type. MedtrCycB-type were divided into two subgroups, including CycB-type (CycB1-B2) and CycB-like type (CycBL1-BL8). The detailed data regarding *M. truncatula* cyclins are provided in Table S2.

### 2.3. Cyclin Gene Structure and Conserved Domain and Motif Analysis

The structure and exon/intron distribution of a gene are largely related to its function. To further study the gene and protein structure of the cyclin gene family, we analyzed the number and distribution of exons in *M. truncatula*, soybean and common bean (Figure S2, Table S3). Cyclin genes in the same type or subgroup had similar exon numbers. All putative legume cyclins could be classified into four major clades, of which the H-type had the largest average number of exons with 9.75, followed by the C-type (9.00), B-type (except CycB-like cyclins (8.87)), J18-type (8.67) and A-type (8.29). The CycB-like cyclins with the smallest average number of exons (1.65) were significantly different from other B-type cyclins, which suggested that they might be functionally specific (Table S3). Moreover, almost all the U-type cyclins had two exons, indicating their structures were completely similar (Figure S2).

A typical cyclin contains an important cyclin_N domain, also named the “cyclin box”, which is found in all known cyclins and is highly conserved. In *M. truncatula* cyclin genes, all members have a cyclin_N domain, but not all members have the cyclin_C domain (Table S2). To elucidate the distribution of the motifs in cyclin proteins and their function, 10 types of motifs and their distribution of legume cyclins were predicted using the MEME program (Figure S2). Our results indicated that cyclins in the same type or subgroup also contained similar motif types. In addition, all putative legume cyclins could be divided into four big groups according to the type of motifs, consistent with the distribution of the exons (Figure S2). There were four major motifs, motif 1, motif 8, motif 7 and motif6 or motif 10, in the A-, B- and SDS-type cyclins of *M. truncatula*, and, of note, motif 10 was identified in CycB-like cyclins, while motif 6 was identified in other A-, B- and SDS-type cyclins. D-type cyclins had four motifs (motifs 4, 5, 9, and 10); C-, H-, L- and T-type cyclins contained three motifs (motifs 2, 8, 10); motif 3 was identified in the U- and J18-type cyclins.

### 2.4. Genome Distribution Across Cyclins on Chromosomes

To analyze the chromosomal distribution of these predicted cyclin genes, we performed searches for position information about these cyclin genes and genetic distances from the three legume plant genome database (Table S3). The results suggested that 57 of 58 *M. truncatula* cyclins, 103 soybean cyclins and 51 common bean cyclins were mapped onto all 8, 20 and 10 chromosomes, except chromosome 11 (Figures S3–S5). The overall distribution of members on the chromosomes was mostly uneven, as *M. truncatula* cyclins were distributed onto chromosomes 1, 2, 3 and 5, with a maximum of 17 on chromosome 3 and a minimum of one (*CycBL-2*) on chromosome 6. Interestingly, all CycB-like cyclins were distributed on chromosome 3, except *CycBL-1* and *CycBL-2* (Figure S3). These results showed that CycB-like cyclins might be tandem duplication events (TDs), providing an explanation for the increase in B-type members. The majority of soybean cyclin genes were mapped onto chromosomes 1, 3, 4, 6, 14 and 17, with more than six members of each. There was only one member on chromosome 16 (Figure S4). In common bean cyclins, most of the members were located on chromosomes 1, 3 and 9, with a maximum of nine on chromosome 9. Cyclin genes were absent on chromosome 4, and there was only one member on chromosome 6 (Figure S5).

### 2.5. Segmental Duplication Event of the M. truncatula Cyclins and Synteny Analysis

**Figure 2:**
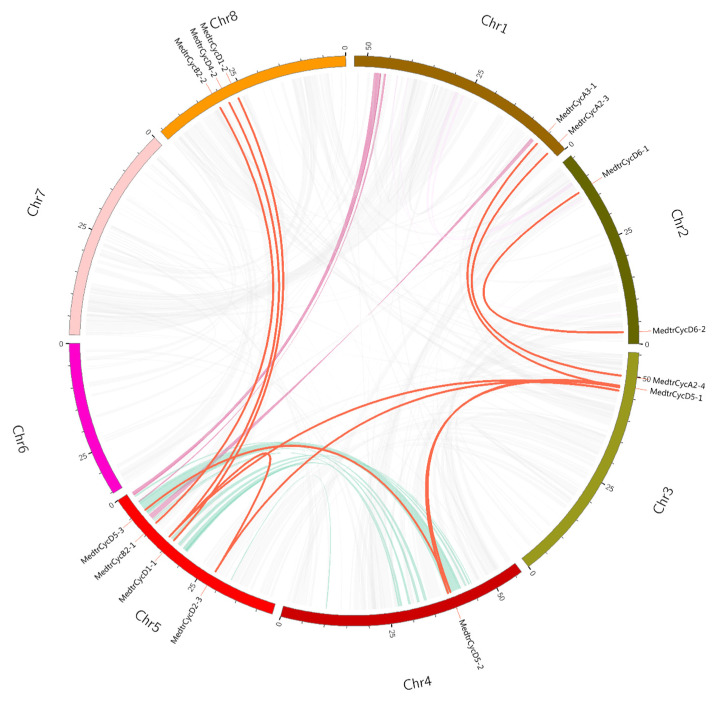
Circos figure for chromosome locations with Mtcyclin segmental duplication links. The red lines indicate segmented duplicated gene pairs.

Segmental duplications lead to duplicated genes through polyploidy, followed by chromosome rearrangements. We performed a series of BlastP searches to understand the gene segmental duplications in cyclins, and the segmental duplicated genes of *M. truncatula* cyclins were identified by MCScanX and CIRCOS. We found a total of 9 colinear gene pairs involving 20 gene members in the *M. truncatula* genome (Figure 2, Table S4). Most of the duplicated gene pairs consist of two cyclin genes and are located on chromosomes 3 and 5; three members of the two duplicated gene pairs were found (MedtrCyclin_Segmetal-5 and MedtrCyclin_Segmetal-8, Table S4).

**Figure 3:**
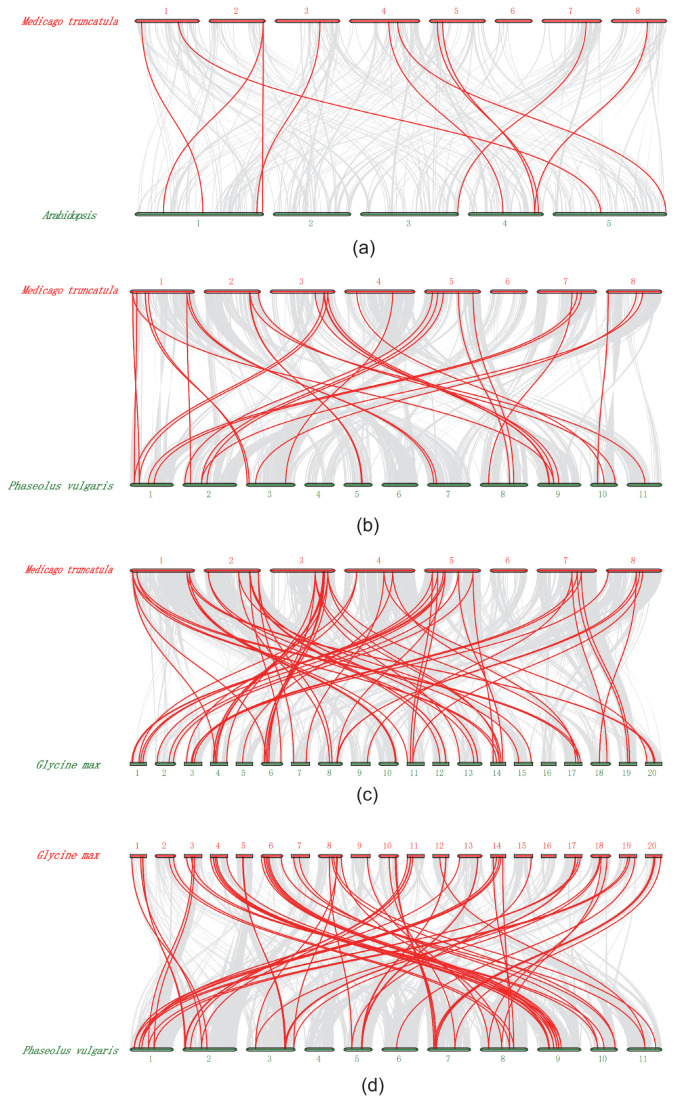
Synteny of cyclin genes in different genome of *M. truncatula*, *Arabidopsis*, soybean and common bean. (**a**) Synteny of *Atcyclin* and *Mtcyclin* gene pairs. (**b**) Synteny of *Mtcyclin* and *Pvcyclin* gene pairs. (**c**) Synteny of *Gmcyclin* and *Mtcyclin* gene pairs. (**d**) Synteny of *Gmcyclin* and *Pvcyclin* gene pairs.

Furthermore, we investigated the synteny of cyclin genes among the *M. truncatula, Arabidopsis,* soybean and common bean genomes (Table S5). A total of only nine syntenic gene pairs were identified between *Arabidopsis* and *M. truncatula*. Similarly, we identified 37 syntenic gene pairs between *M. truncatula* and soybean, 39 between soybean and common bean, and 27 between *M. truncatula* and common bean (Figure 3, Table S5). We found that most *Medicago* and common bean cyclin genes might have more than one orthologues in soybean. Notably, some *M. truncatula* cyclin members had more than two orthologues in soybean, and, interestingly, almost all (33 of 39) cyclin genes in common bean had two orthologous genes in soybean (Table S5). These results suggested that soybean, a tetraploid plant, likely contained twice the number of cyclins observed for *M. truncatula* and common bean. 

### 2.6. Prediction Analysis of Cis-Acting Elements within M. truncatula Cyclin Genes

**Figure 4:**
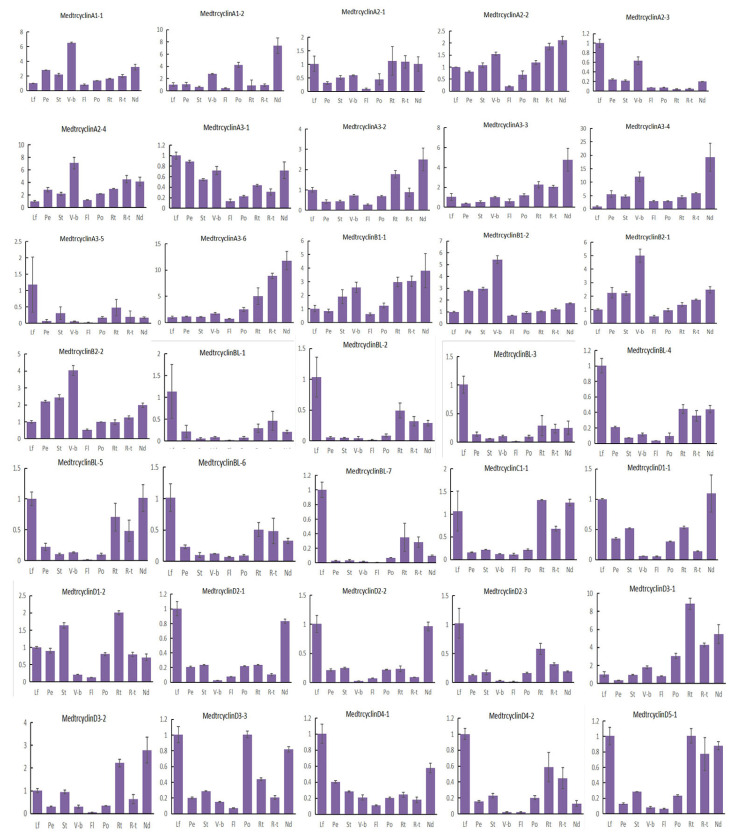

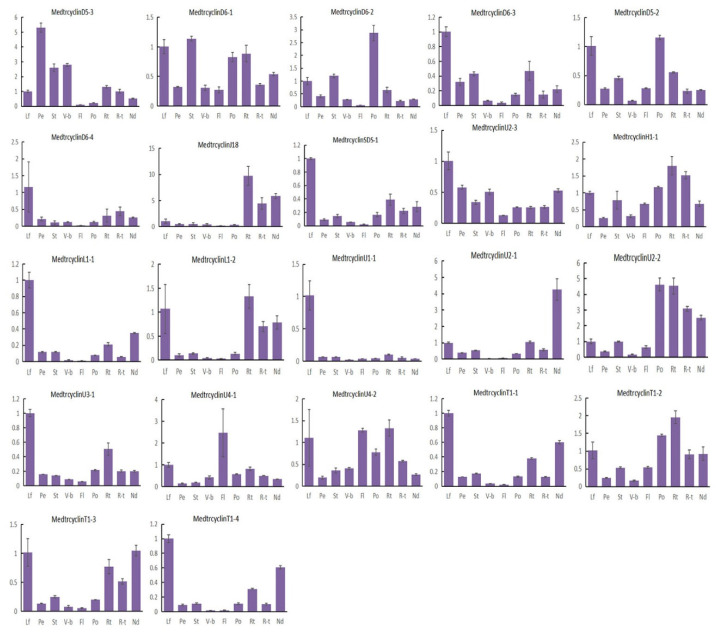
Expression levels of *Mtcyclins* in various tissues. Expression profiles of *Mtcyclin* genes were analyzed in nine different tissues: leaf (L), petiole (Pe), stem (St), vegetive bud (V-b), flower (Fl), pod (Po), root (Rt), root tip (R-t), and nodule (Nd). The data are from three independent biological replicates, and error bars indicate standard deviations.

Specific cis-element motifs can be recognized by transcription factors and participate in gene expression regulation. To further study the potential regulatory mechanisms of *M. truncatula* cyclins in a diversified biological process, particularly in plant hormones, meristem development, cell cycle regulation and pathogen infection, 2.0 kb upstream sequences from the translation start sites of cyclin genes were submitted to the PlantCARE database to detect cis-elements [44]. A total of 12 known cis-elements were searched for analysis, including the TATA-box and CAAT-box, which are the most important and basic elements and were present in all cyclin members and 80.0% of the cyclin promoters (Figure S6, Table S6). ABRE, as-1, HD-Zips and W-box, which participate in hormonal responses, were present in 19.4, 12.5, 1.0 and 9.2% of cyclin promoters, excluding the TATA-box and CAAT-box. The CAT-box, G-box, as-1, GT1-box, MYB (v-myb avian myeloblastosis viral oncogene homolog) binding site (MBS), stress-response element (STRE) and W-box cis-elements respond to high salt, dehydration, low temperature, light and osmotic pressure related to biotic or abiotic stress responses (Table S6). We found that most cyclin promoters contained these elements (79.0%), indicating that these genes might respond to biotic or abiotic stress responses. For instance, many transcription factor (TF) family members bind to G-boxes, such as one of the largest basic helix–loop–helix (bHLH) and basic Leu zipper (bZIP) families [45], and regulate the function of their target genes. Previous research has indicated that the G-box, a cis-acting element of *CHS15,* is essential for floral and root-specific expression and as a tissue-specific regulatory element in French bean [46]. Twenty-five of the *cyclin* genes, including *MedtrCycD3-1, MedtrCycD3-2, MedtrCycD5-1* and *MedtrCycL1-2,* were preferentially expressed in roots (Figure 4) and found to contain the G-box element related to root-specific expression in their promoter regions. These results implied that these four genes might be good candidates to regulate root development (Table S6). In addition, the as-1 element, an important cis-element in plant biotic or abiotic stress responses, especially in the plant defense response, enhanced the expression of putative plant protective genes in response to xenobiotic chemical stress [47,48]. Our results showed that 39 cyclins, including 14 genes that were highly and specifically expressed in nodules (Figure 4), contained the as-1 element (Table S6).

### 2.7. Expression Patterns of M. truncatula Cyclin Genes in Different Tissues

To investigate the possible roles of the *M. truncatula* cyclins, the expression of 57 cyclin genes in *M. truncatula* were determined by quantitative real-time PCR results in 9 various tissues, including leaves, petioles, stems, vegetative buds, flowers, pods, roots, root tips and nodules. Our results indicated that *M. truncatula* cyclin genes showed diverse expression profiles in different tissues (Figure 4). The majority of the cyclins were expressed in all tissues tested, with various expression levels. Further analysis suggested that approximately more than half of the cyclin genes (33) were highly expressed in the leaves of *M. truncatula*. Among them, CycA3;5, CycBL;1, CycBL;3, CycBL;7, CycD4;1, CycD6;4, CycSDS;1, CycL1;1 and CycU1;1 were highly and specifically expressed in leaves. In addition, 4 members were highly expressed in petioles, 13 in stems, 13 in vegetative buds, 3 in flowers, 12 in pods, 36 in roots, 20 in root tips and 31 in nodules. It is worth noting that some cyclin members were preferentially expressed in some specialized tissues. For example, only CycD5;3, CycU4;1 and CycD6;2 were specifically expressed in petioles, flowers and pods, respectively. It is possible that they play an important role in the growth and development of these organs in *M. truncatula*. In addition, it is noted that CycU4;1 might be involved in meiosis. Moreover, CycA3;2, CycA3;3, CycA3;6, CycD3;1, CycD3;2, CycJ18 and CycU2;2 were highly and specifically expressed in roots, roots tips and/or nodules. Moreover, CycJ18, a very divergent gene, exhibited a unique expression pattern, suggesting that it also has specialized functions in roots and nodules, as it is specifically expressed in roots as *Arabidopsis* CycJ18 [15].

Our results also indicated that cyclins in the same types exhibited very similar expression patterns, suggesting possible functional redundancy between the highly similar genes. In general, most members of the A-, B- and D-types were highly expressed in leaves, petioles, stems, vegetative buds, pods, roots and nodules, suggesting that these genes might be important for the mitotic cell cycle and/or mitotic growth, which is in accordance with prior studies [49]. Members of L-, T- and U-types were highly expressed in leaves, flowers, roots and nodules. Additionally, few members were highly expressed in more than one tissue, for example, CycU2; 2 was highly expressed in pods, roots, root tips and nodules. Specifically, the A-type members CycA1;1, CycA1;2, CycA2;2, and CycA3;4 were highly expressed in vegetative buds and nodules, indicating that they might play important roles in nodule development [42]. The CycB-like type, including CycBL;1, CycBL;2, CycBL;3, CycBL;4, CycBL;5, CycBL;6 and CycBL;7 genes, as the specific clade of *M. truncatula*, have the similar expression pattern and were highly expressed in leaves, roots and nodules. In addition, some segmental duplication gene pairs have a similar expression tendency in tissues, for example, CycB2;1, CycB2;2, and some D-type cyclins (e.g., CycD1;1, CycD1;2, CycD2;1 and CycD2;3). However, some segmental gene pairs were expressed differently in various tissues (Figure 4, Table S4).

### 2.8. Expression Patterns of M. truncatula Cyclins under Sinorhizobium Medicae Infection

**Figure 5:**
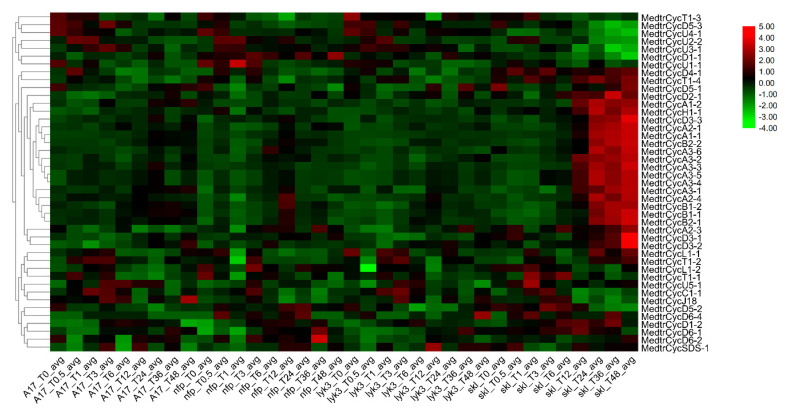
Heatmap of expression for *Mtcyclins* under rhizobium infection. The genome-wide RNA-seq data of *M. truncatula* were obtained from the Query genes expression website. The expression data of cyclins in wild-type (WT) A17, mutants with absent or decreased nodulation (Nod) factor sensitivities (i.e., nodulation factor perception (*nfp*) and lysine motif domain-containing receptor-like kinase 3 (*lyk3*), respectively) and the Nod factor-hypersensitive mutant (sickle, *skl*). This data set encompasses nine time points, allowing observation of the symbiotic regulation of diverse biological processes with high temporal resolution.

To further study the expression profiles of *M. truncatula* cyclins in legume-rhizobium symbiosis under *S. medicae* infection, a database ([50] http://pages.discovery.wisc.edu/~sroy/Medicago_symbiosis_transcriptome/query.php) was used to identify a differential expression matrix of the corresponding members and build an expression heatmap [44]. In this treatment, one wild type (WT) and three mutants seedings were inoculated with *S*. *medicae*. A17 is a wild type (WT) with a normal nodulation phenotype. The mutants *nfp* [51] and *lyk3/hcl-1* [52] show no or decreased Nod factor sensitivities, respectively, while Nod factor-hypersensitive and ethylene-insensitive mutants (sickle, *skl* [53]) are supersensitive to *Rhizobium* and supernodulation [50]. The expression profiles of 44 cyclins were determined in *Medicago* roots during *S. meliloti* infection (Table S7, Figure 5). Among them, 23 cyclin genes were highly and specifically expressed in the *skl* mutant beginning at 12 h after rhizobium infection (Table S8) and peaking at 48 h, showing that they may also specifically respond to ethylene (ET) signals during the rhizobial infection [50]. Approximately half of them (11) were A-type cyclins, which indicated that the A-type was a major cyclin member playing a significant role in the nodulation process in *Medicago*. Simultaneously, their expression degree was comparatively high in WT, followed by *lyk3*, but they were hardly expressed in the *nfp* mutant (Figure 5), which shows that they have the same respond with the Leucine-rich repeat receptor-like kinases (LRR-RLKs ) in *M. truncatula* [44]. These results indicated that these 23 gene members might be closely related to the symbiotic process of rhizobia and might have a certain function in the cell cycle process of nodulation. Combining the expression profiles in different tissues with those in roots after *S*. *medicae* infection, we also found that approximately 9 of 22 cyclins (*CycA1;2*, *CycA3;2*, *CycA3;3*, *CycA3;4*, *CycA3;6*, *CycB1;1*, *CycD3;1*, *CycD3;2* and *CycD5;1*) were preferentially expressed in roots and nodules and responded specifically to Nod factors and ethylene (ET) signals in nodulation. Particularly, *CycA1;2, CycA3;3*, *CycA3;4*, *CycA3;6*, *CycD3;1* and *CycD3;2* were specifically expressed in roots and/or nodules and might be the candidate genes that participate in the symbiosis and Nod factor-mediated cell cycle of nodulation activation (Figure 5). For the segmental cyclin genes, we found A-type cyclins (*CycA2;3* and *CycA2;4, CycA3;1* and *CycA3;2*) and B-type cyclins (*CycB2;1* and *CycB2;2*) were highly responded to in the *skl* mutant, starting at 12 hpi (Figure 5, Table S4).

**Figure 6:**
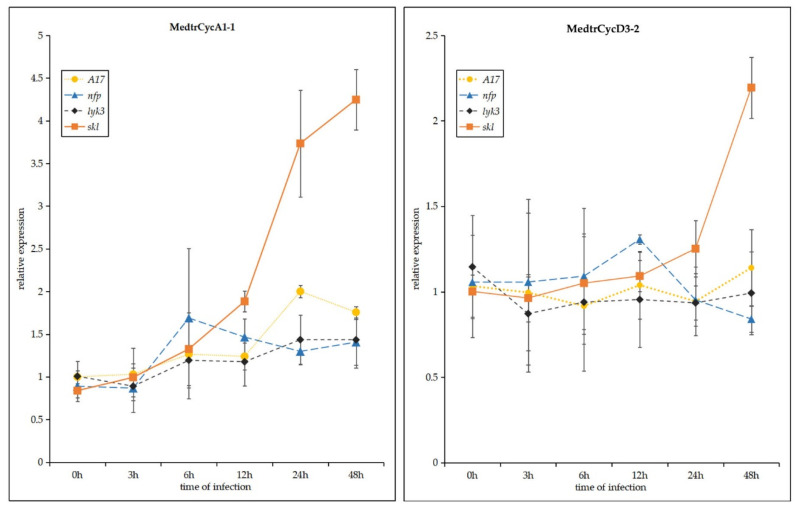
Expression profiles of *Mtcyclin* genes were analyzed after *Sinorhizobium meliloti* infection in four *M. truncatula* genotypes: wild-type A17, *nfp*, *lyk3* and *skl* mutants. Values in the line graphs show average trimmed mean of M component (TMM) counts normalized to cv Jemalong A17 at 0 hpi. Error bars represent standard error (SE) calculated from three independent biological replicates.

In order to further investigate the expression profile of these genes expressed specifically or highly in the *skl* mutant after 12 h of *S. meliloti* infection (Figure 4 and Figure 5), we studied the expression levels of the *Mtcyclin* genes by qRT-PCR (Figure 6). In the *skl* mutants, it was observed that the expression levels of *CycA1;1 and CycD3;2* were significantly increased, starting at 12 hpi. The expression level of *CycA1;1* in the *skl* mutant was much higher than that in WT A17, *lyk3* and *nfp* at 12, 24 and 48 hpi, and *CycD3;2* was highly expressed at 24 and 48 hpi, which was similar with the RNA-seq expression profile (Figure 6).

## 3. Discussion

Cyclins complex with CDKs to control the activity, substrates and subcellular localization of CDKs and play an extremely important role in cell division of the cell cycle in plants [1,12]. They interact with CDKs and other proteins to participate in almost the entire mitosis process, and play crucial roles in the growth and development of animals and plants [54,55]. In this study, a total of 58, 103 and 51 cyclin genes in the *Medicago*, soybean and common bean genomes were identified (Table S2). Phylogenetic analysis indicated that all putative legume cyclins were classified into ten types (A-, B-,C-, D-, H-, L-, T-, U-, SDS-, and J18-types), sharing the same types with *Arabidopsis*, whereas the D-type was the largest clade in the legume species, and the CycB-like types, including CycBL;1-8, were specific to *Medicago,* lacking any clear homologues in *Arabidopsis*, common bean and soybean. Likewise, F-type cyclins, lacking clear homologues in *Arabidopsis*, were unique to rice, and Q- and Z-types were defined as new putative types of poplar cyclin genes [19,20]. D-, CycJ18-, T-, SDS-, and U-type cyclins are not specific to *Arabidopsis* but are also present in other plants [15]. For conserved domains, our study indicated that most of the A-, B-, and D-types contained both cyclin_N and cyclin_C domains; however, each of the U- and T-types in all putative cyclins had only cyclin_N without cyclin_C domains, as also reported in *Arabidopsis*, maize, rice and poplar [15,18,19,20]. Additionally, together with the structures and motifs of these cyclins, the same types of cyclins in *Arabidopsis* and legume plants were located in the same clades and shared high sequence similarity. The types in the same big clade contained similar motifs and numbers of exons, such as A-, B- and SDS-types and C-, H-, L-, and T-types. It is noteworthy that all of the cyclins could be identified into four major clades: the first clade included the A-, B- and SDS- types; the second clade was the D-type; the third clade contained the C-, H-, L-, and T-types; and the forth clade contained the U- and J18-types (Figures S1 and S2).

Cyclins were found to have a maintained diversity distribution on all legume chromosomes, whereas several numbers of gene members were on different chromosomes, although no members were distributed on chromosome 4 in common bean. If two genes shared more than 70% similarity, they were identified as tandem duplications or segmental duplications [56]. In our study, we found nine pairs of segmental duplication genes in *Medicago*, most of which (six gene pairs) were present in the D-type, and two gene pairs were located in the A-type (Table S4). The D-type group had the largest number of members, indicating that segmental duplications were one of the reasons for the expansion of the subfamily members [44,57]. In the cyclin gene family, most soybean members had two or more genes with a collinear relationship observed for a corresponding cyclin gene of *Medicago* or common bean (Table S5). In addition, a pair of genes with collinearity between different genomes was almost in the same family, and their sequence, gene structures and motifs were similar, indicating a completed evolutionary separation before species evolution, as well as a certain degree of functional similarity and redundancy. Cis-acting element analysis indicated that five *Medicago* cyclins contained HD-zip elements participating in the ethylene signal response. Among them, CycA2-4 and CycT1-4 cyclins were specifically responsive in the *skl* mutant (an ethylene-insensitive mutant) during *S*. *medicae* infection (Figure 5, Table S7), suggesting that the HD-zip element in these cyclins might have particular roles in their function.

In *Medicago*, a new sub-type of cyclins known as CycB-like type (CycBL;1-8) was identified, with no corresponding homologues in other plant species (Figure 1). The gene structure and motif analysis revealed that these eight cyclin genes clearly differed from other B-type cyclins. Most of them had only one exon and contained motif 10, identified in the D-, C-, L-, and T-types, rather than motif 6 detected in other B-type cyclins, suggesting that the functions of CycB-like type cyclins might be similar to A-, B- and D-types (Figure S2, File S1). Further analyses are needed to determine the exact functions of these *Medicago*-specific CycB-like type cyclins. Genome distribution analysis demonstrated that six of eight B-like-type cyclins were located on chromosome 3 (Figure S3). Cis-acting element prediction analysis revealed the presence of the as-1 element, an important cis-element in plant biotic or abiotic stress responses, especially the plant defense response; this was identified in CycBL;1, CycBL;3, CycBL;5, CycBL;6, CycBL;7 and CycBL;8 cyclins (Table S7). Expression profiles indicated that they were expressed in leaves, roots and nodules; however, they did not largely respond in the Nod factor-hypersensitive mutant *skl* during *S*. *medicae* infection, suggesting that they might play roles in the division of nodule cells rather than the response to nodulation activation of Nod factor induction (Figure 4 and Figure 5). Our results also indicate that CycB-like type cyclins in the same types exhibited similar expression patterns, suggesting a possible functional redundancy between them.

Two additional cyclins, SDS and CycJ18, were quite isolated from the others and treated as two separate classes in Arabidopsis [15,58,59]. Phylogenetic evolution, gene structure and motif analyses suggested that the SDS-type was clearly related to the A- and B-types, and the J18-type to the D-type. A similar phenomenon has also been found in tomato [60]. Rice lacks C- and J18-type cyclins, but it contains the additional F-type and larger D-type cyclins, which may function as C- and J18-type cyclins [18]. Similarly, there are no SDS- and J18-types in poplar, but they have homologous Z- and Q-types that cluster with the B-type and D-type clades, respectively [20]. These results suggest that cyclins in plants play more complicated and diverse roles in cell cycle progression, which is consistent with the results obtained for *Arabidopsis* [61]. Expression profiles showed that CycJ18 cyclin of Medicago as well as poplar cyclin CycQ1;1 and CycZ1;1 were specifically expressed in roots, stems or nodules, suggesting that these genes might have specific functions in target tissues and organs. Besides, *Arabidopsis* cyclins like A-, B- and SDS-type cyclins also play important roles in meiosis [62]. However, all A- and B-type cyclins were lowly expressed in the flowers, but U-type CycU4;1 and CycU4;2, expressed specifically and highly in the flowers, might participate in the meiosis of the sexual life cycle in *M. truncatula*. However, their expression profiles and functions need to further investigate in the future.

Nodule development requires two signaling events [42]. First, flavonoids are exuded by the root [63] and interact with bacterial regulatory NodD proteins to induce the expression of nodulation genes in rhizobia [64]. Second, Nod factors, which are produced and secreted by nodulation gene products, elicit cell division in the root pericycle and cortex and form a novel meristem that develops into the nodule primordium [42,65]. The formation of nodule organogenesis starts with the nodule primordium, including nodule initiation and development. During the development of nodules, some cyclin genes were activated and expressed throughout almost the entire process. In *M. sativa*, A-type cyclins, especially *CycA2*(*Medsa;CycA2;2*)*,* participated in the G0/G1 transition of nodule organogenesis cell cycle processes [38,42]. In our study, all A-type cyclins in *M. truncatula*, except *MedtrCycA2;2*, were specifically and highly expressed from 12 to 48 h in the mutant *skl* during *S*. *medicae* infection. Moreover, *MedtrCycA1;2, MedtrCycA3;3, MedtrCycA3;4* and *MedtrCycA3;6* were highly expressed in vegetable buds, roots and nodules. Previous research has indicated that the expression of *MedtrCycA3;1*, an A-type cyclin homologous to *Arabidopsis CYCA3;1*, is increased during rhizobial infection and that it exhibits very high expression in meristematic tissues [43]. Similarly, we found other CycA3 cyclins, namely, *MedtrCycA3;3*, *MedtrCycA3;4* and *MedtrCycA3;6*, which were specifically and highly expressed in roots, root tips and nodules, as well as the mutant *skl,* during rhizobia infection, which is consistent with the proposed role of CYCA3 proteins in mitosis [66,67,68]. Another important type, D-type, showed conserved regulation of G1 phase progression in plants and animals. In *Medicago*, *CycD3* genes might have a particular role in developmental cell cycle programs, such as either the recruitment of G0 cells and/or the endoreplication cycles in division-arrested cells [42]. In *M. truncatula*, *MedtrCycD3;1* and *MedtrCycD3;2* were specifically and highly expressed in roots, root tips or nodules and mutant *skl* during rhizobia infection. *Medsa;CycD3;1*, as a D-type cyclin, was induced in the G1 phase of the cell cycle after activation of the A2-type cyclin [39], which suggested that *MedtrCycD3;1* and *MedtrCycD3;2* might play particular roles in the G1 phase of the cell cycle during nodule initiation and development like *Medsa;CycD3;1*(*ascycMs4*). Moreover, we noted that cyclins located on chromosome 3 were CycB-like, A- and D-type cyclins, whereas all A- and D-type cyclins were specifically expressed in the *slk* mutant during rhizobia infection and clustered together (Figure 5 and Figure S3). Excluding A- and D-type cyclins, B-type cyclins, together with the A-type, interacted with CDKs (cyclin-dependent kinases) and regulated cell cycle progression during the G2 phase and G2/M transition [37,40,49]. The expression of three B-type cyclins (MedtrCycB1;1) was specifically increased in the *skl* mutant during rhizobial infection, suggesting the involvement of B-type cyclins in nodule initiation and prominent function in the late stage of nodule development in legume plants such as *Medicago* (*M. truncatula and M. sativa*) and lupin [37,42,69]. Additionally, the U-type cyclin MedtrCycU2;1 was also specifically expressed in nodules, which might be a new candidate cyclin that performs particular roles in nodule development.

In this study, detailed phylogenetic analysis of these three legume plant cyclins and expression profiles of *Medicago* cyclins provided useful information for future research. Those *Medicago* cyclins with specific expression patterns could be the focus of functional studies to determine their possible roles in specific tissues and symbiosis. However, cyclin proteins play various roles in the cell cycle process of plant growth and development, and functions such as response to biotic or abiotic stress should be researched in the future.

## 4. Materials and Methods

### 4.1. Arabidopsis Cyclin Family and Three-Species Genome Resources

According to the research, a total of 50 cyclin genes were identified in *Arabidopsis* based on the domains [15]. The amino acid sequences of all Atcyclin family members were acquired from *Arabidopsis_thaliana*.TAIR10 in Phytozome v12.1 (https://phytozome.jgi.doe.gov/pz/portal.html) [70]. Cyclins were identified in three Leguminosae species: *M. truncatula*, soybean (*G. max*), and common bean (*P. vulgaris*). The genomic sequences, coding sequences, and peptide sequences of these three Leguminosae species annotated genes were downloaded from Phytozome v12.1 (https://phytozome.jgi.doe.gov/pz/portal.html) [70].

### 4.2. Identification of Cyclin Genes in the M. truncatula, Soybean and Common Bean Genome

To identify cyclin genes, the method of hidden Markov model searching was used to search the domains [71]. Putative genes were initially identified by searching the cyclin_C domain and cyclin_N domain (LRR1(PF00560), LRR5(PF13306)) obtained from Pfam database version 32.0 (http://pfam.xfam.org) [72] with HMMER v3.1 in Linux. Proteins containing detectable cyclin_N alone or both cyclin_N and cyclin_C domains were regarded as cyclins [15]. Then, we again screened the results using an E-value less than 0.001, and only the first transcript was used. Furthermore, these sequences were filtered by the description and functional annotation in Phytozome v12, followed by analysis with SMART v9.0 (http://smart.embl-heidelberg.de) [73], Pfam database version 32.0 (http://pfam.xfam.org/) and NCBI-CDD v3.18 (https://www.ncbi.nlm.nih.gov/cdd) to ensure the presence of a cyclin_C domain and a cyclin_N domain. Finally, following repeated alignment with known Atcyclin family members, a phylogenetic tree was constructed, and the inaccurate sequence was deleted.

### 4.3. Multiple Sequence Alignments, Phylogenetic Tree Analysis and Classification of Cyclin Genes in M. truncatula

Multiple sequence alignments were performed by using ClustalW and Muscle in MEGA7.0 [74] based on the amino acid sequences of the cyclin_N domain. Unrooted phylogenetic trees were constructed for *M. truncatula* cyclins and *Arabidopsis* or Fabaceae species: *M. truncatula*, soybean and common bean and *Arabidopsis* together with the neighbor-joining (NJ) method [75]. The nodes were tested by bootstrap analysis with 1000 replicates, and the tree with the highest likelihood was selected for further analysis. The evolutionary distances were computed using the p-distance method [3] and are presented as units of the number of amino acid differences per site. Then, the iTols v5.0 (https://itol.embl.de/itol.cgi) [76] and Evolview v2.0 (http://www.evolgenius.info/evolview) website and AI CS6 software were used to modify the phylogenetic tree.

### 4.4. Gene Structure and Protein Conserved Motif Analysis of Cyclins

The positions of the mRNA and positions and numbers of introns, exons, and the untranslated region (UTR) regions were batch-extracted from genomic annotation of the gff3 file in the Linux system. Combined with the phylogenetic tree, the exon–intron structures of the Leguminosae cyclin genes were identified using the GSDS v2.0 website (http://gsds.cbi.pku.edu.cn/index.php). The protein conserved motifs were predicted with MEME-v4.12.0 [77], and the chart was drawn using TBtools [78] and AI software.

### 4.5. The Chromosome Location, Duplications and Synteny Analysis

To locate the *cyclin* genes on the chromosome, the locations of genes and chromosome length were acquired from genomic annotation of the gff3 file in Linux. All Leguminosae *cyclins* were mapped onto chromosomes for each species based on their physical positions, respectively. The charts showing the chromosome location were drawn with gene-map v2.0 (http://mg2c.iask.in/mg2c_v2.0.). Tandem duplications were characterized as multiple members of this gene family occurring within neighboring intergenic regions [79]. The segmental duplicated genes of Mtcyclins were identified by MCScanX [80] and CIRCOS [81] with the genomic protein sequences, which were blasted against themselves. The synteny of cyclins with different genomes including *Arabidopsis, M. truncatula*, soybean and common bean were also mapped by MCScanX with collinearity in Linux.

### 4.6. Cis-Acting Element in Mtcyclin Gene Promoter Analysis

The upstream sequences (2.0 kb) of the *cyclin* genomic DNA were retrieved from the genome sequence data in Linux and then submitted to PlantCARE v1.0 (http://bioinformatics.psb.ugent.be/webtools/plantcare/html/) [82] to identify cis-acting elements. Finally, in this study, we selected 12 elements related to hormone induction, such as abscisic acid (ABA)-responsive elements, ethylene-responsive elements, stress-responsive-like defense, low-temperature responsive elements, and light. The website GSDS v2.0 (http://gsds.cbi.pku.edu.cn/index.php) was used to draw the map.

### 4.7. Expression Analysis

#### 4.7.1. Plant Material and qRT-PCR Analysis of Mtcyclin Genes

Four *M. truncatula* genotype seeds, wild-type cv Jemalong A17, *nfp* (C31), *lyk3* (*hcl-1*, B56) and *skl* (*skl1-1*), were scarified, germinated and grown in aeroponic tanks (caissons) or pots under long day conditions: light/dark photoperiod, 16/8 h at 21 °C; humidity, 75%; light intensity (photosynthetically active radiation, PAR), 300 μmol.m^−2^.s^−1^ (HQL 400 De Luxe mercury vapor bulbs, Osram, 24600 lux) [53]. Five-day-old plantlets were inoculated with *Sinorhizobium medicae* ABS7M. *S. medicae* ABS7M was grown in tryptone yeast (TY) medium supplemented with 6 mM.L^−1^ calcium chloride and 10 μg mL^−1^ tetracycline at 28 °C for 48 h. The culture was washed three times and finally resuspended in 10 mL sterile distilled water to an OD600 of 1.0, which was used to inoculate aeroponic caissons containing 10 L of low-nitrogen aeroponic medium [55]. Root samples were collected at 0, 3, 6, 12, 24, and 48 hpi. Three independent biological replicates per time point and genotype were collected and immediately frozen in liquid nitrogen for RNA isolation.

To study the expression profiles of *Mtcyclins* in various tissues and under rhizobium infection, total RNAs were isolated from roots, root tips, nodules, stems, leaves, petiole, vegetables buds, flowers, and pods using Qiagen RNeasy kits (Qiagen, Beijing, China). A cDNA template was generated from equivalent quantities of RNA using a Quantitect Reverse transcription kit (Qiagen). Quantitative RT-PCR was performed on an ABI 7500 Fast Real Time System (Davis, California, America) using Power SYBR Green PCR Master Mix (Applied Biosystems), and the primers used are listed in Table S1. The PCR program consisted of an initial denaturation step (20 s at 50 °C) and a polymerase activation step of 10 min at 95 °C, followed by 40 cycles of 15 s at 95 °C and 1 min at 60 °C. The relative expression level of the gene was calculated using the ΔΔCt method and normalized using ubiquitin carrier protein (Medtr3g062450/TC17644) mRNA [53].

#### 4.7.2. Expression Analysis of Mtcyclin Genes during Rhizobium Infection

The expression data for rhizobium infection were acquired based on the query gene expression ([55], http://pages.discovery.wisc.edu/~sroy/Medicago_symbiosis_transcriptome/query.php), including WTs, mutants with absent or decreased Nod factor sensitivities (i.e., nodulation factor perception (*nfp* [50]) and lysine motif domain-containing receptor-like kinase 3 (*lyk3* [51]), respectively), and an ethylene (ET)-insensitive and Nod factor-hypersensitive mutant (sickle, *skl*) [52,53]. For each mutant, transcriptional changes occurring in the roots of *M. truncatula* at 0, 0.5, 1, 3, 6, 12, 24, 36, and 48 h (a total of 9-time gradients) after inoculation with *Rhizobium* were acquired. Heatmaps were generated with TBtools [78]. The means were derived from three repeated expression values.

## 5. Conclusions

In this study, a genome-wide analysis of legume *cyclin* gene families was performed, and a total of 58, 103 and 51 members were identified in the *M. truncatula,* soybean and common bean genome. They were classified into 10 types (A-, B-, C-, D-, H-, L-, T-, U-, SDS- and J18-types) according to the *Arabidopsis cyclin* genes. We analyzed the phylogeny, classification, gene structures and motifs, positions, duplications, cis-acting elements and expression profiles of cyclin genes in *M. truncatula,* common bean and soybean. We found that the CycB-like type were unique to *M. truncatula* and lacking in other legume plants. Further investigation on the expression patterns in various tissues and under rhizobial infection in *M. truncatula* suggested that some candidate cyclin genes might have specific roles in Nod factor-mediated cell cycle activation and nodule development during symbiosis. Our study provides wide insights to the cyclin gene family in legume plants, especially *M. truncatula*. Some cyclins have specific functions in target tissues and cell cycle of organ activation and development, which should be identified in the future.

## Figures and Tables

**Table 1:** Cyclins’ content in *Arabidopsis*, *M. truncatula*, soybean and common bean.

Cyc-Type	*Arabidopsis*	*M. truncatula*	Soybean	Common Bean
CycA	11	12	20	10
CycB	11	12	13	6
CycC	2	1	2	2
CycD	9	17	40	18
CycH	1	1	2	1
CycL	1	2	2	1
CycT	5	4	8	4
CycU	7	7	14	7
CycJ18	1	1	1	1
CycSDS	1	1	1	1
Total members	49	58	103	51

## Supplementary Materials

The following are available online at https://www.mdpi.com/1422-0067/21/24/9430/s1, Figure S1: Unrooted phylogenetic tree of cyclins. The N domain sequences from all putative cyclins were aligned by ClustalW, and the phylogenetic tree was constructed using MEGA 7.0 by neighbor joining with 1000 bootstrap replicates. Figure S2: The exon/intron, untranslated region (UTR) organization and motifs of all cyclin genes. Exons are represented by yellow boxes and introns by black lines. UTR regions of some genes are also indicated using green boxes. The relative sizes of exons, introns and UTR can be estimated by the length of boxes or lines. Ten motifs were predicted. Figure S3: Genomic distribution of cyclin genes across *Medicago truncatula* chromosomes. The positions of cyclins are acquired in A17 MtrunA17r4.0 genome. Figure S4: Genomic distribution of *cyclin* genes across soybean chromosomes. The positions of cyclins are acquired in A17 MtrunA17r5.0 genome. Figure S5: Genomic distribution of *cyclin* genes across common bean chromosomes. The positions of cyclins are acquired in A17 MtrunA17r5.0 genome. Figure S6: The predicted cis-acting element in *Mtcyclin* promoters. The 2.0 kb upstream sequences of the all *Mtcyclin* genomic DNA sequences were retrieved. Table S1: The primers used for quantitative real time RT-PCR. Table S2: List of the identified *cyclin* genes in *M. truncatula,* soybean and common bean. The ID, gene code, gene length, physical position on chromosome, number of exons/introns/UTRs, length of amino acids, and number of kinase domains for each cyclin gene in this study are included. Table S3: The physical chromosomal positions of *Mtcyclin* genes and chromosome length of M. *truncatula,* soybean and common bean. Table S4: List of segmental duplicated cyclin gene pairs in M. *truncatula*. Table S5: List of synteny gene pairs in *M*. *truncatula* and *Arabidopsis*, *M. truncatula* and soybean, *M. truncatula* and common bean, and soybean and common bean genome. Table S6: The data of the cis-acting element in *Mtcyclin* promoters. Table S7: Expression profiles for *Mtcyclins* under rhizobium infection (http://pages.discovery.wisc.edu/~sroy/Medicago_symbiosis_transcriptome/query.php). Table S8: List of *Mtcyclins* highly and specifically expressed under rhizobium infection after 12 hours. Members in yellow shading were specifically expressed in the roots, nodule and *skl* mutant. (http://pages.discovery.wisc.edu/~sroy/Medicago_symbiosis_transcriptome/query.php). File S1: The sequence alignment of B-type cyclins.
