# Supplementary material for: Genome-Wide Analysis of the Cyclin Gene Family and Their Expression Profile in Medicago truncatula
Source: Int J Mol Sci. 2020 Dec 11;21(24):9430. doi: 10.3390/ijms21249430 (PMC7763586; doi:10.3390/ijms21249430)
Supplement: Supplementary file 1 [file ijms-21-09430-s001.zip › Supplementary files/File S1 sequence alignment of B-type.docx]

Figure 1. Protein sequences alignment of the B-like cyclins with the CycB1-1 cyclin.The darkgreen box means the position of the motif 6 in CycB1-1 or motif 10 in CycB-likes. The red box means another differences between their proteins.


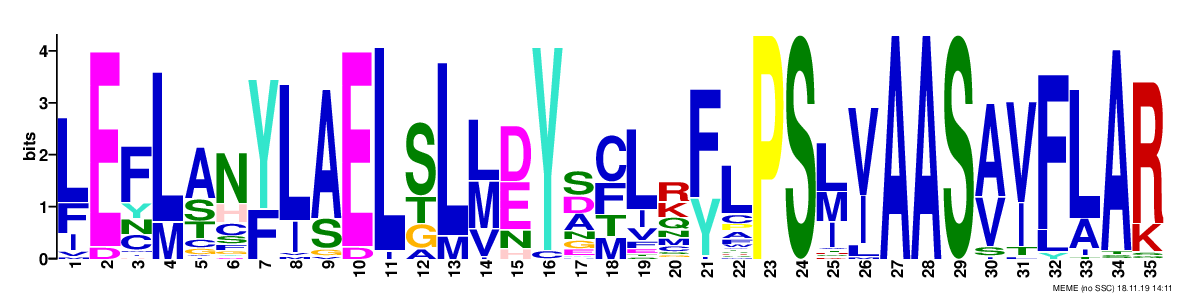


Figure 2.The sequences of the motif 6


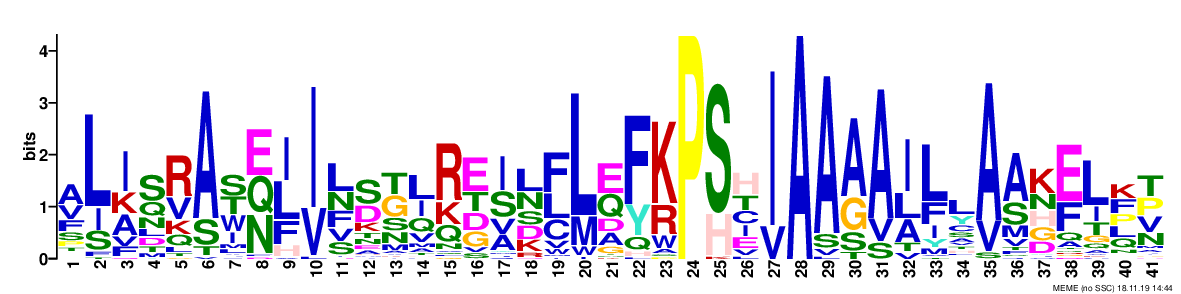


Figure 2.The sequences of the motif 10
