# Supplementary figures and images for: Genome-Wide Analysis of the Cyclin Gene Family and Their Expression Profile in Medicago truncatula
Source: Int J Mol Sci. 2020 Dec 11;21(24):9430. doi: 10.3390/ijms21249430 (PMC7763586; doi:10.3390/ijms21249430)

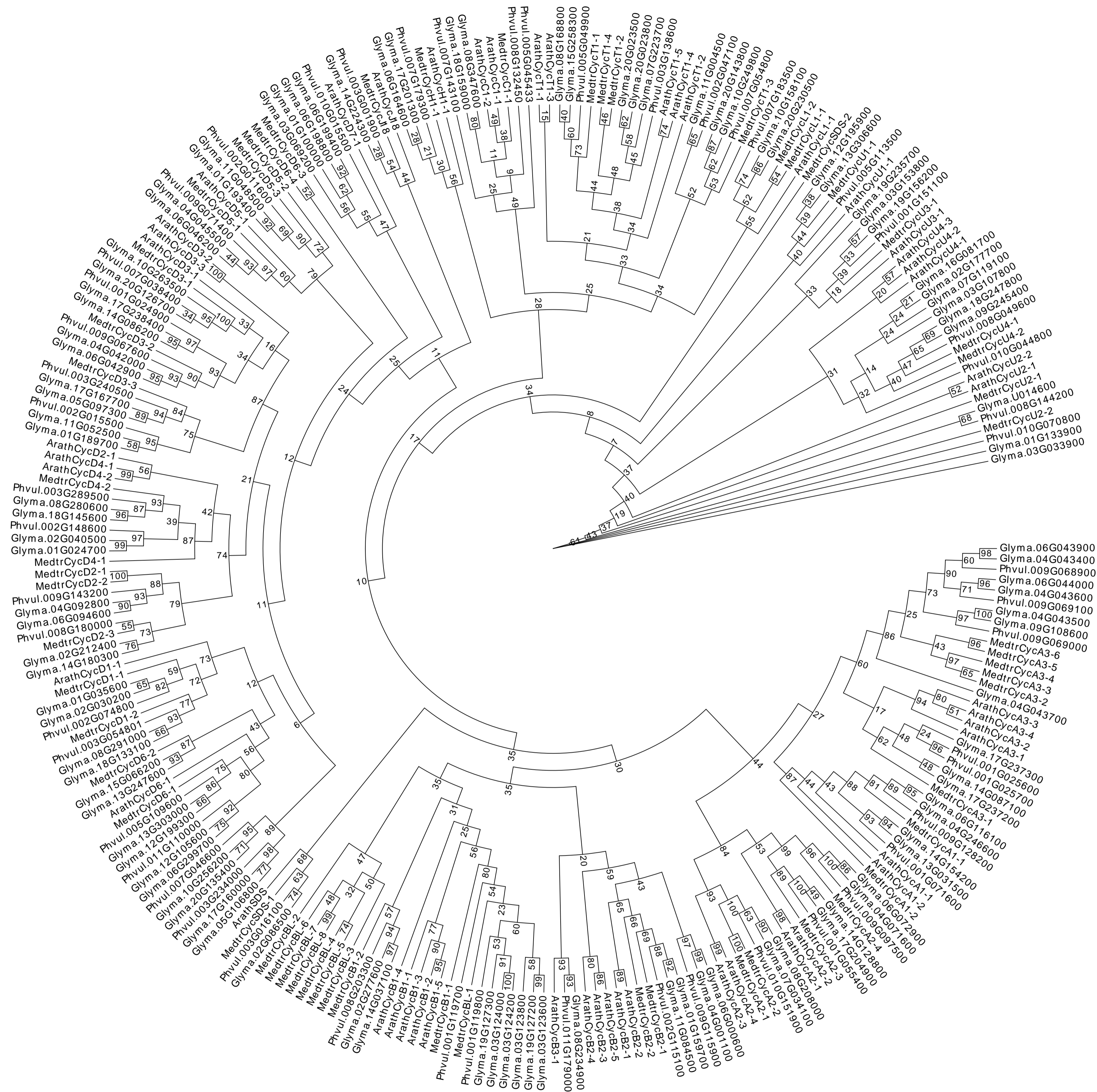

Supplement: Supplementary file 1 [file ijms-21-09430-s001.zip › Supplementary files/Figure S1 AT-MT-GM-PV_MLtree.pdf]

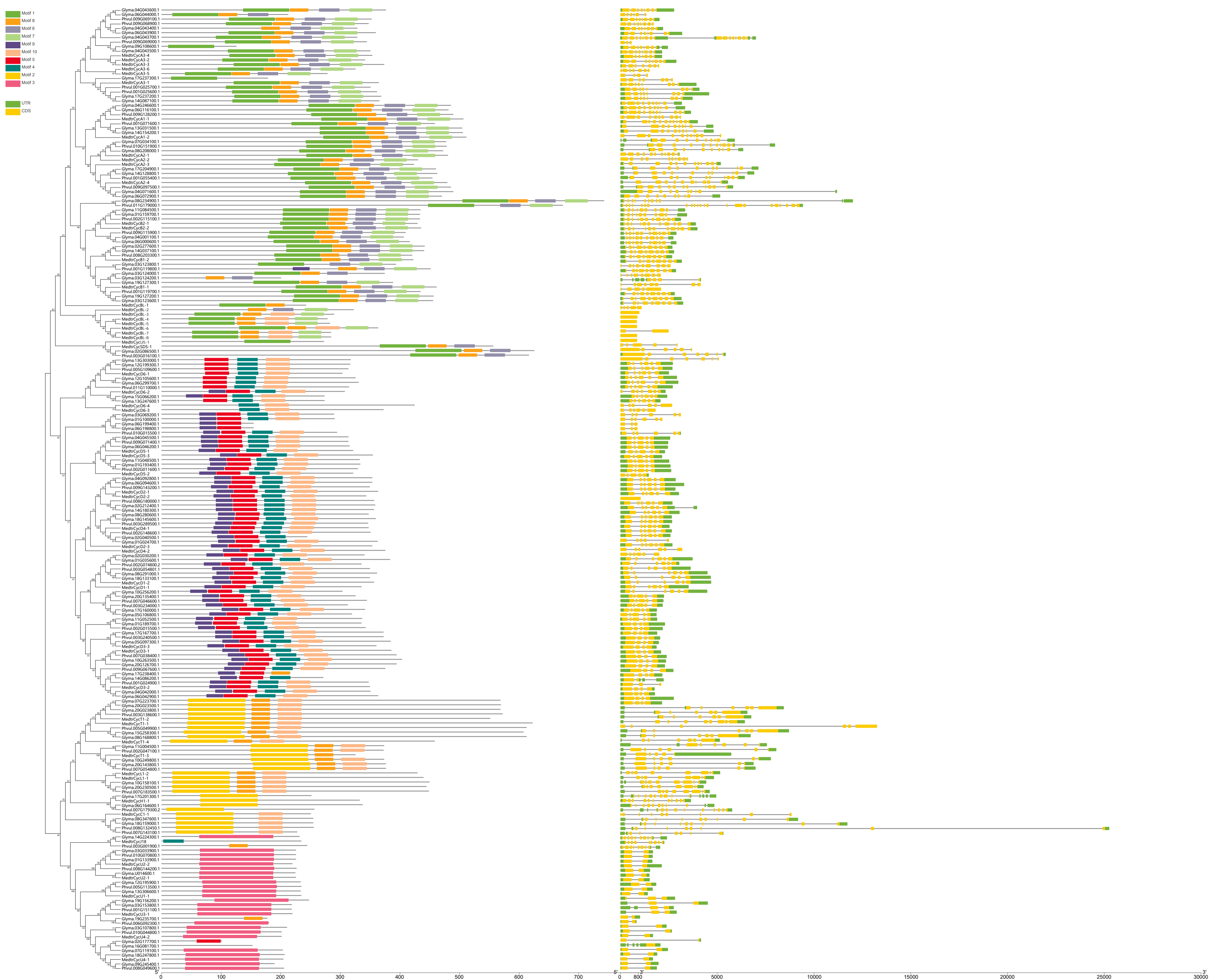

Supplement: Supplementary file 1 [file ijms-21-09430-s001.zip › Supplementary files/Figure S2 Structure and motif of MT_GM_PV.pdf]

**chr1**

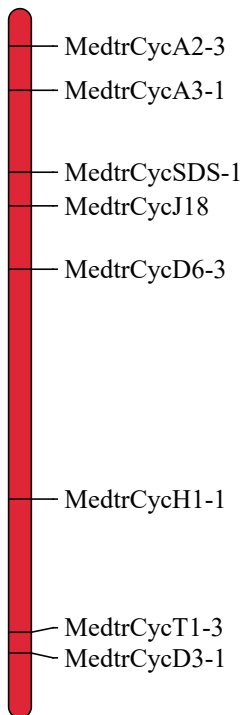

**chr2**

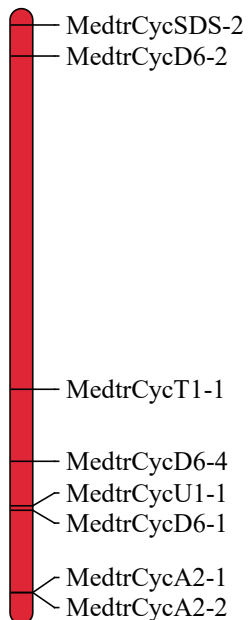

**chr3**

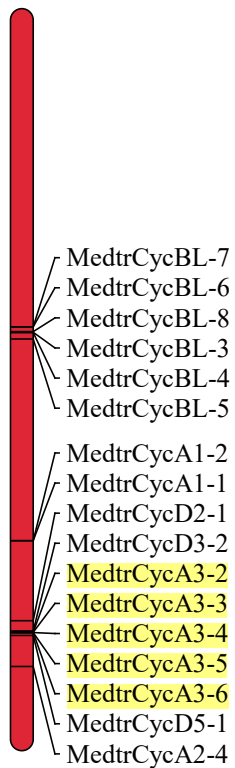

**chr4**

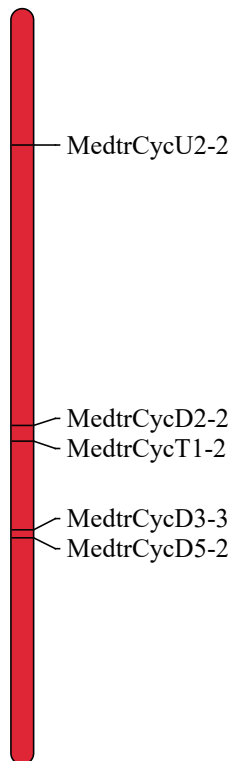

**chr5**

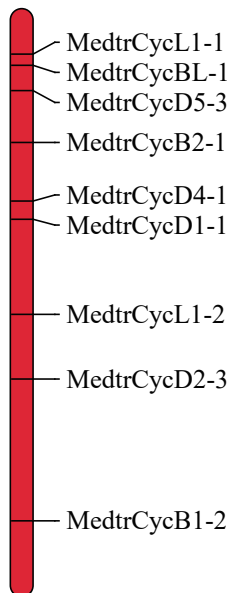

**chr6**

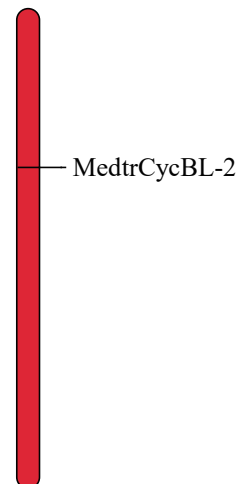

**chr7**

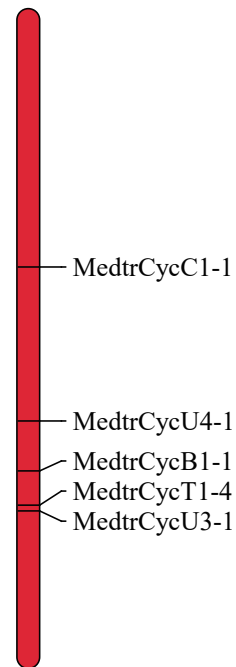

**chr8**

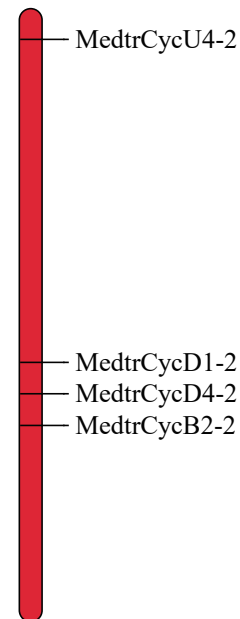

**Gene-location**

Supplement: Supplementary file 1 [file ijms-21-09430-s001.zip › Supplementary files/Figure S3 MT_map-to-chr.pdf]

chr1

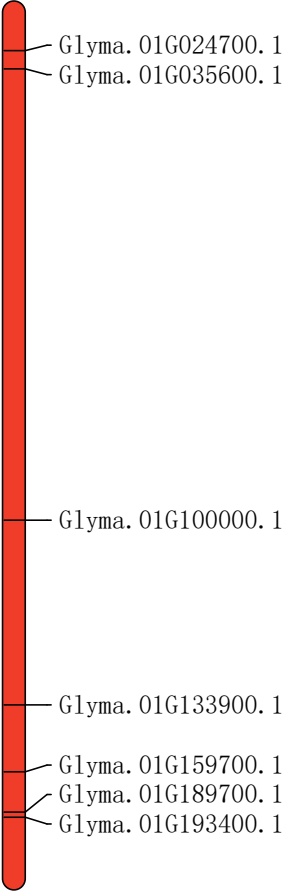

chr2

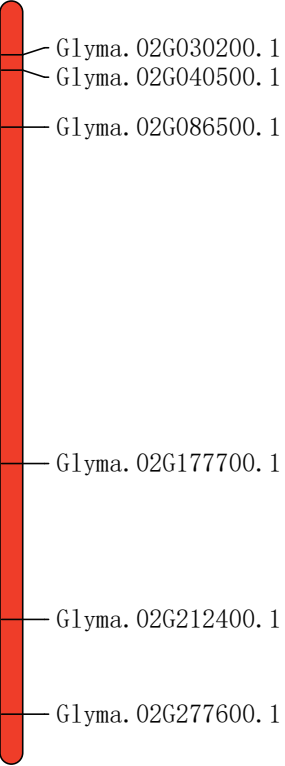

chr3

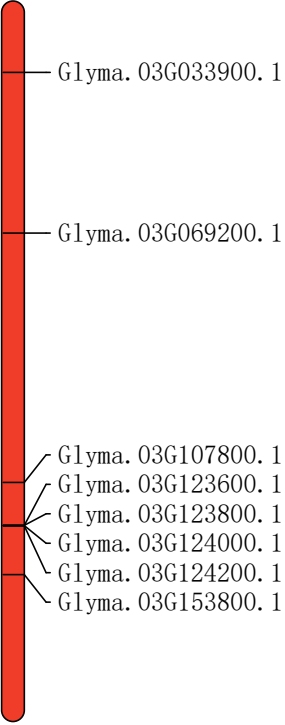

chr4

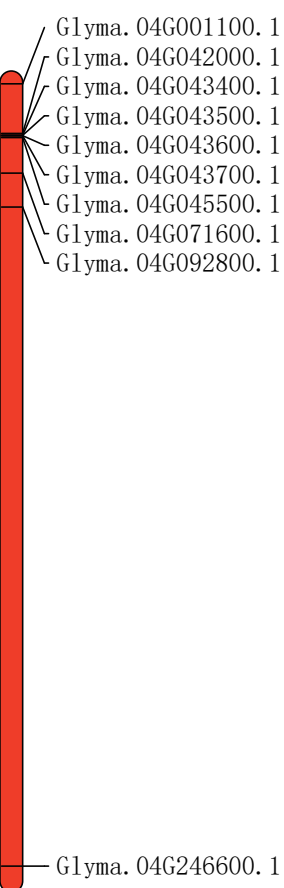

chr5

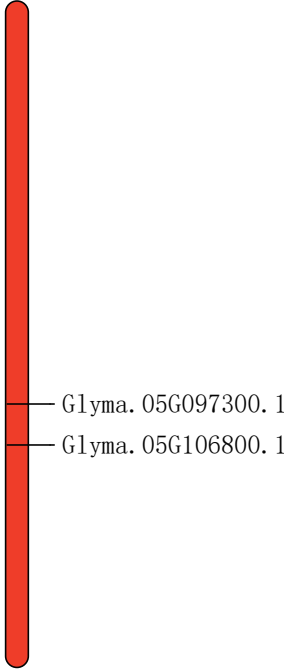

chr6

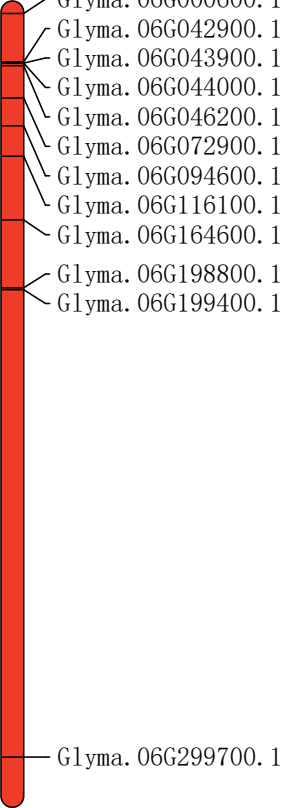

chr7

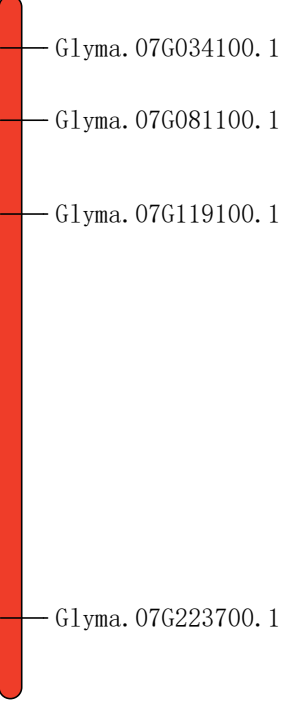

chr8

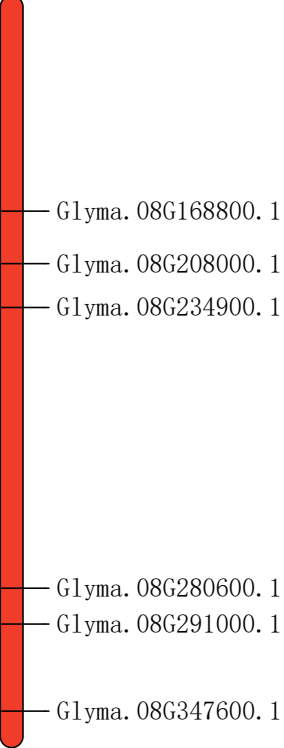

chr9

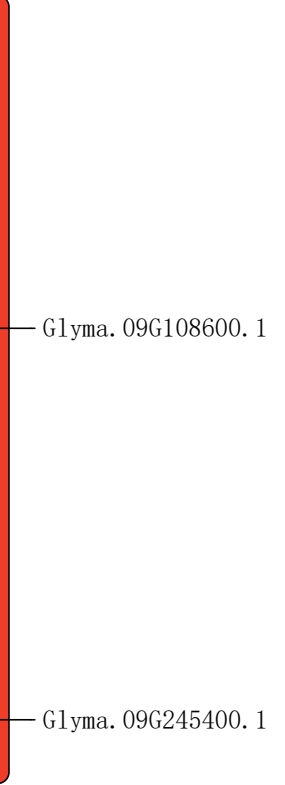

chr10

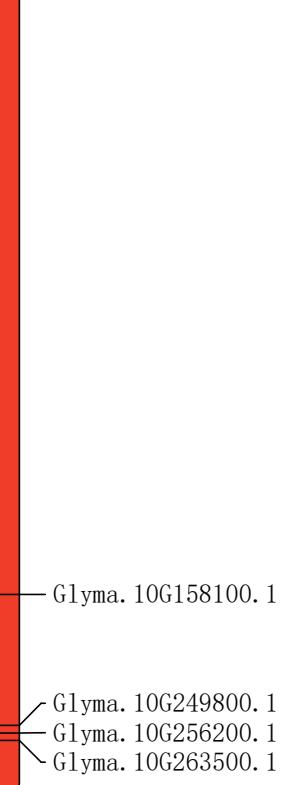

chr11

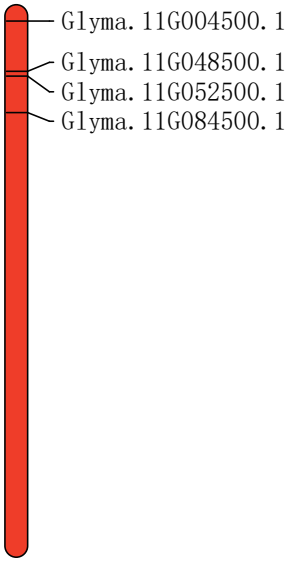

chr12

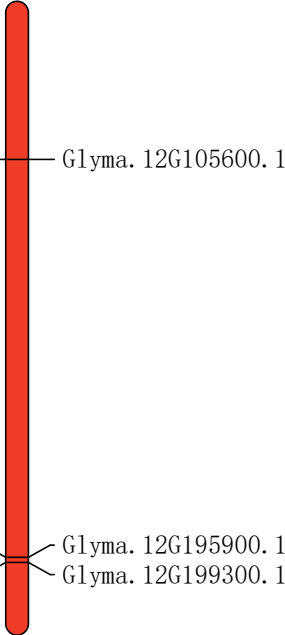

chr13

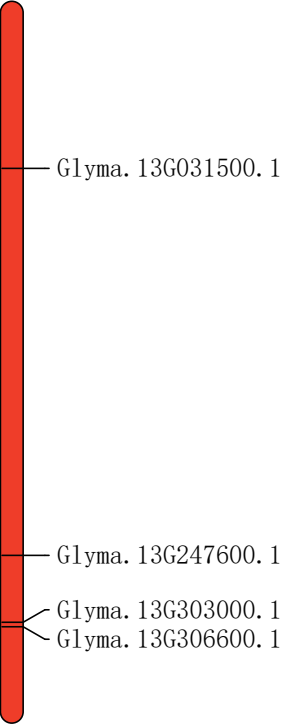

chr14

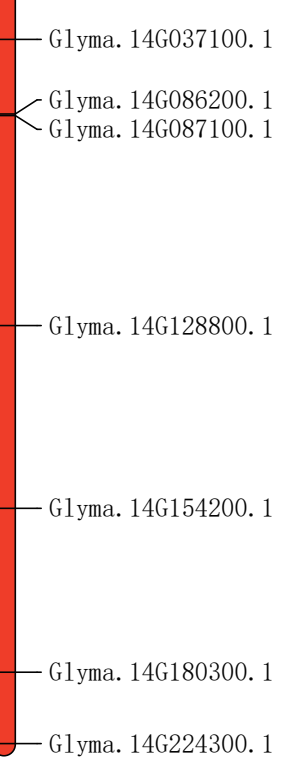

chr15

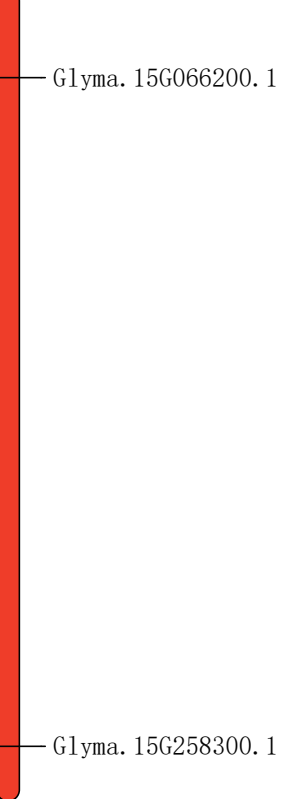

chr16

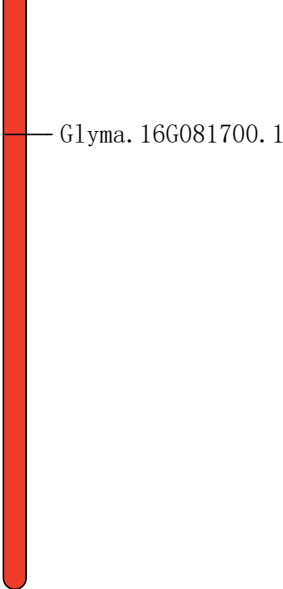

chr17

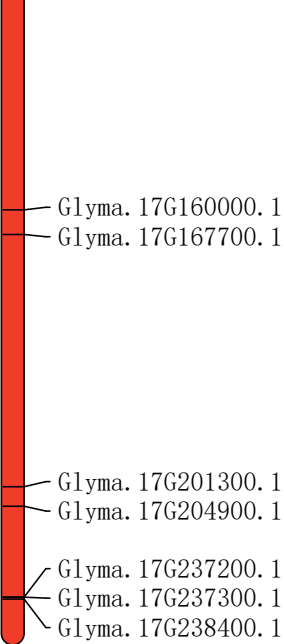

chr18

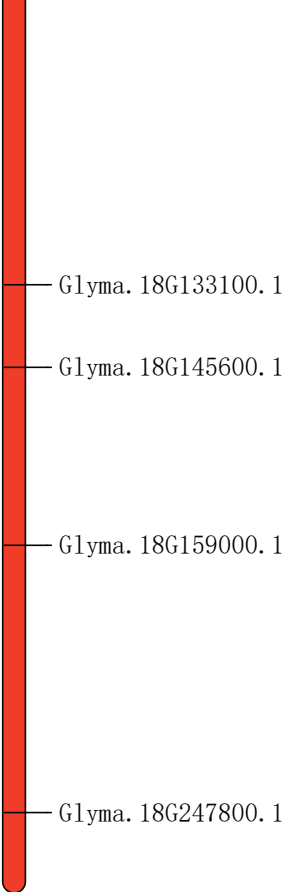

chr19

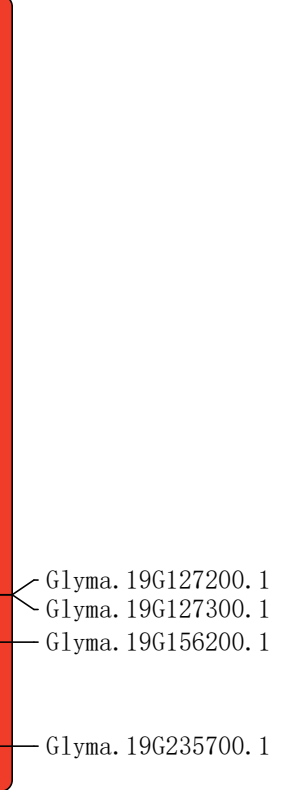

chr20

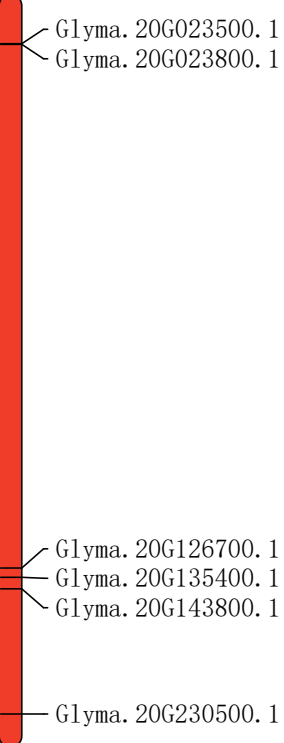

Supplement: Supplementary file 1 [file ijms-21-09430-s001.zip › Supplementary files/Figure S4 GM_mapchart.pdf]

chr1

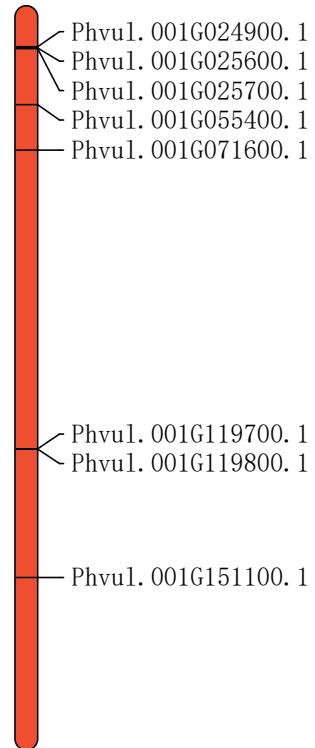

chr2

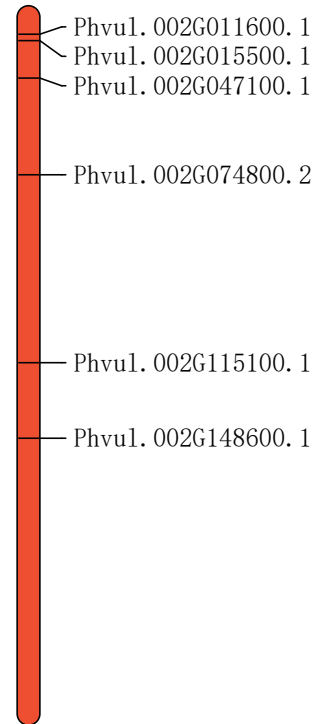

chr3

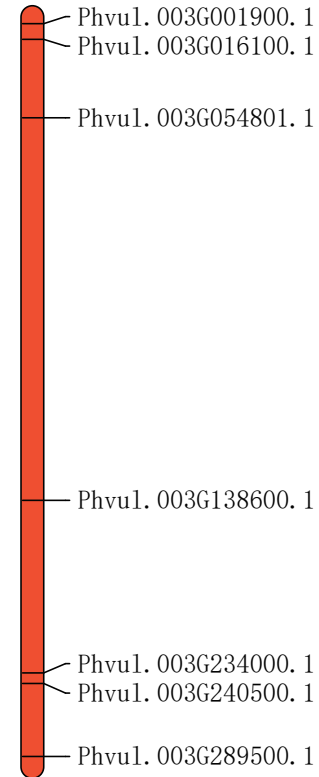

chr5

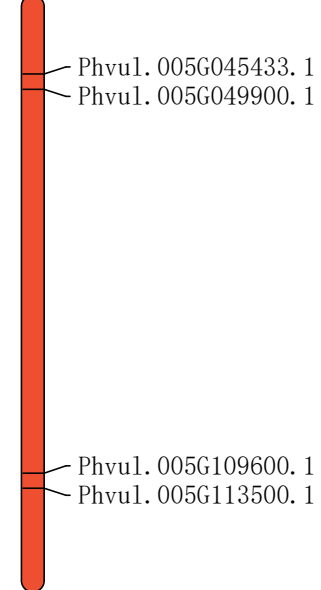

chr6

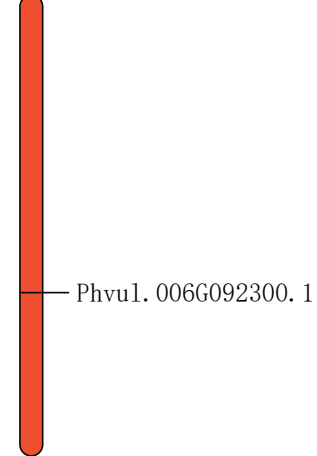

chr7

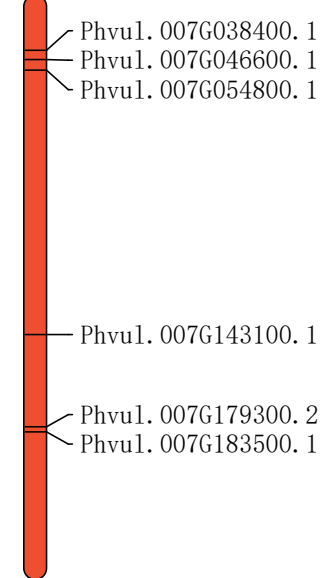

chr8

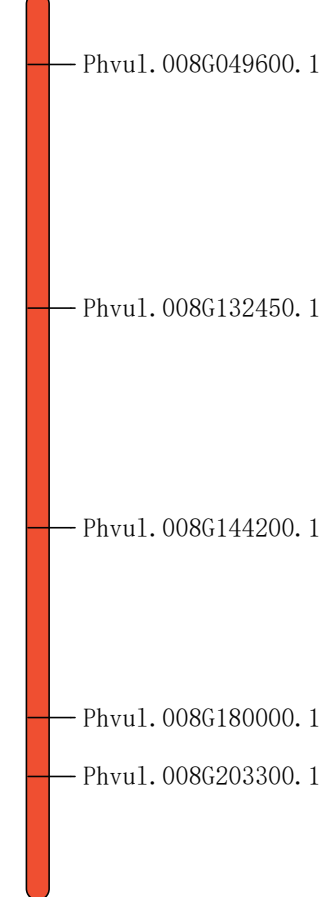

chr9

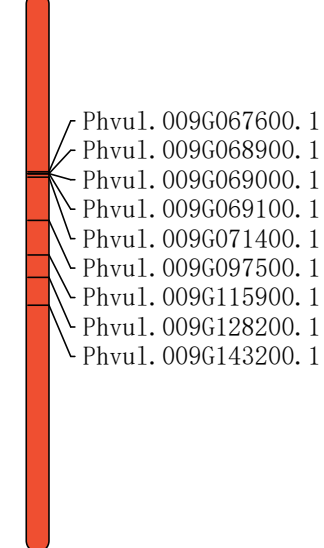

chr10

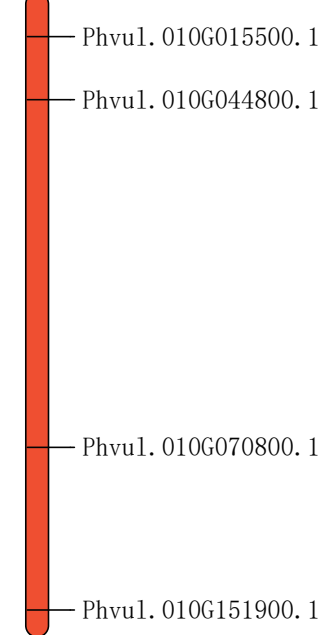

chr11

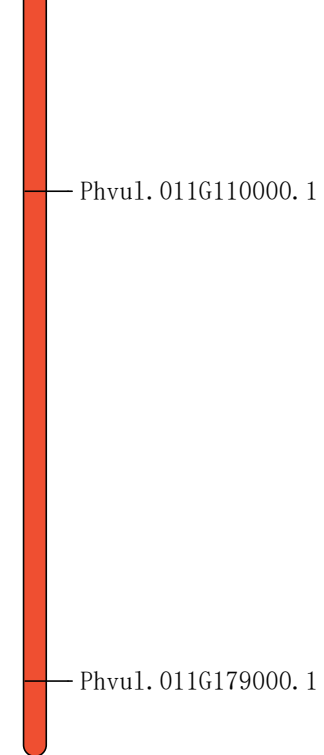

Supplement: Supplementary file 1 [file ijms-21-09430-s001.zip › Supplementary files/Figure S5 PV_mapchart.pdf]

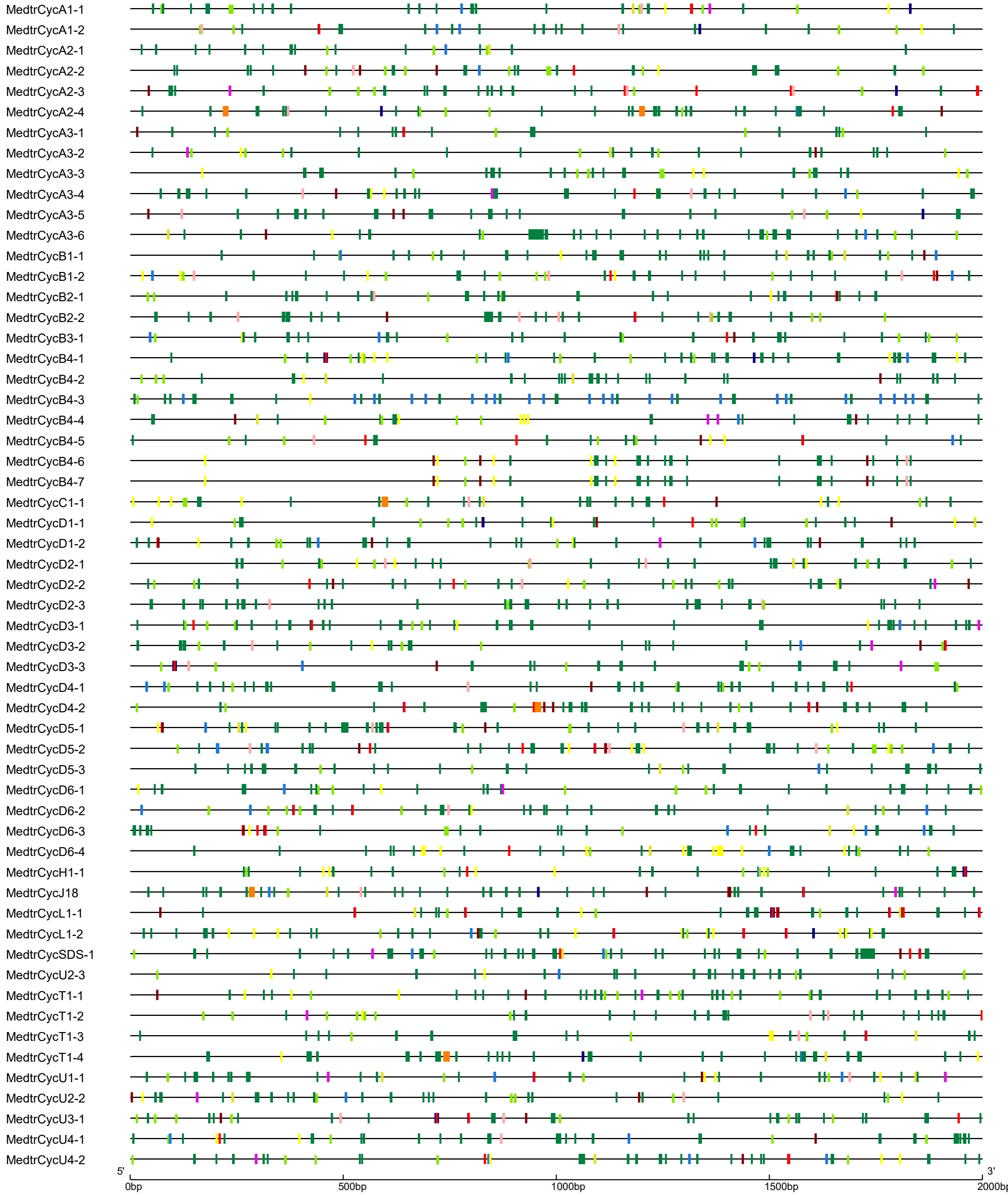

Legend:

— Exon   CAAT-box   TATA-box   CAT-box   G-box   GT1-motif   MBS   STRE   HD-Zip   as-1   ABRE   W

Supplement: Supplementary file 1 [file ijms-21-09430-s001.zip › Supplementary files/Figure S6 promoter.pdf]
